# Characterization of genetic context of *bla*_KPC_ in *Pseudomonas aeruginosa*

**DOI:** 10.3389/fmicb.2025.1666175

**Published:** 2025-11-14

**Authors:** Mingxiao Chen, Tingting Deng, Xiaobin Li, Runcheng Zhou, Zhangshu Gao, Die Zhou, Jianqiang Zhu, Jingjie Li, Xin Chen, Minling Wang, Rui Zhang, Qiang Zhou

**Affiliations:** 1 Department of Clinical Laboratory Medicine, The Second Affiliated Hospital, Guangzhou Medical University, Guangzhou, China; 2 Department of Pulmonary and Critical Care Medicine, Zhuhai People’s Hospital (The Affiliated Hospital of Beijing Institute of Technology, Zhuhai Clinical Medical College of Jinan University), Zhuhai, China; 3 School of Medicine, Foshan University, Foshan, China; 4 Department of Nephrology, Zhuhai People’s Hospital (The Affiliated Hospital of Beijing Institute of Technology, Zhuhai Clinical Medical College of Jinan University), Zhuhai, China

**Keywords:** *Pseudomonas aeruginosa*, carbapenem-resistant, *bla*_KPC_-positive plasmid, regional evolution, transposon

## Abstract

The rising prevalence of *bla*_KPC_-harboring *Pseudomonas aeruginosa* (KPC-PA) is a significant threat to public health. In the study, we identified a carbapenem-resistant *P. aeruginosa* (CRPA; PAE3) from a patient with pneumonia infection. The resistance phenotype was analyzed using the broth microdilution method. Whole-genome sequencing was performed to sequence type (ST), resistance genes, plasmid replicon, and the genetic environment of the *bla*_KPC-2_ gene. The other 75 *Pseudomonas aeruginosa* (PA) isolates with chromosome- or plasmid-borne *bla*_KPC_ gene were used to analyze the mobilized characterization of *bla*_KPC_ gene in PA from a global perspective. The result revealed PAE3 belonged to ST463 and exhibited multidrug resistance, which is the first report of PA harboring a chromosome-borne *bla*_KPC-2_ gene located within a Tn*1721*-like transposon in South China. In total, 76 KPC-PA strains were identified from 7 countries, representing 6 distinct *bla*_KPC_ variants—*bla*_KPC-2_, *bla*_KPC-3_, *bla*_KPC-33_, *bla*_KPC-87_, *bla*_KPC-90,_ and *bla*_KPC-113_—with the majority located within transposons such as Tn*1403*, Tn*1721*, Tn*4401*, or Tn*6296*-like elements. A total of 63 *bla*_KPC_-positive plasmids evolved into different phylogenetic clades in China and other countries, indicating clonal transmission and regional evolution. The dissemination of *bla*_KPC_ is primarily observed in three distinct forms in ST463 KPC-PA in China: (1) plasmid-mediated transfer associated with Tn*6296*-like transposon; (2) Tn*1721,* and (3) the IS*26*-IS*Kpn27*-*bla*_KPC_-2-IS*Kpn6* structural arrangement, which is integrated into both plasmid and chromosome. In contrast, Tn*4401* is commonly observed in Europe and the United States, predominantly emerging in ST235. This study represents the valuable systematic classification of the transposons associated with the spread of *bla*_KPC_ and offers new insights into the mobilized genetic platforms that contributed to the global dissemination of carbapenem resistance in PA.

## Introduction

*Pseudomonas aeruginosa* (PA) is an opportunistic pathogen that thrives in individuals with weakened immune systems, particularly those in intensive care units (ICUs) (Botelho et al., 2019; Forero-Hurtado et al., 2023). This versatile bacterium is responsible for a range of infections, including pneumonia, urinary tract infections, and bloodstream infections, and is increasingly difficult to treat due to its remarkable resistance to multiple classes of antibiotics (Forero-Hurtado et al., 2023; Reyes et al., 2023). Among these, the resistance to carbapenems—powerful broad-spectrum antibiotics used as a last line of defense—poses a significant therapeutic challenge (Li et al., 2024). Carbapenem-resistant PA develops primarily through two mechanisms: (1) the alteration of outer membrane permeability through loss of porin *OprD*, enhancing antibiotic efflux (*MexAB-OprM* and *MexXY–OprM*) and mutation of target penicillin-binding proteins (PBPs); (2) the acquisition of carbapenemase genes, which encode enzymes capable of hydrolyzing and inactivating carbapenems (Aurilio et al., 2022; Qin et al., 2022; Tenover et al., 2022). These resistance genes are frequently found on mobile genetic elements (MGEs), including plasmids, transposons, integrons, and genomic islands, which facilitate the horizontal transfer of resistance between bacteria (Rozwandowicz et al., 2018; Yoon and Jeong, 2021). To date, several families of carbapenemases associated with MGEs have been identified in PA (Hammoudi Halat and Ayoub Moubareck, 2022).

One of the most concerning is the *Klebsiella pneumoniae* (KP) carbapenemase (*bla*_KPC_), a class A carbapenemase that has emerged as a major contributor to resistance worldwide (Hu et al., 2024a; Li et al., 2015; Wang et al., 2024). The *bla*_KPC_ variants are prevalent in diverse Gram-negative bacilli, predominantly *K. pneumoniae* (73.8%), followed by *Enterobacter hormaechei* (7.1%), *Escherichia coli* (6%), and *Enterobacter cloacae* (5.5%), but are rare in PA (Ding et al., 2023). Although most linked to *K. pneumoniae* (especially the pandemic ST258 clone), *bla*_KPC_ in PA remains a significant threat (Wang et al., 2024; Yu et al., 2018). The *bla*_KPC_ gene has been identified in various PA sequence types (STs), including ST463, ST235, ST654, ST1006, and ST1212. Notably, ST463 PA gained attention due to its association with particularly severe infections and higher mortality rates (Li et al., 2024). The ST463 *bla*_KPC_-positive PA (KPC-PA) has shown a numerical dominance and regional prevalence in China, particularly in Zhejiang Province. ST463 is known for its increased virulence and its ability to acquire a diverse range of MGEs, facilitating the spread of various resistance genes, including carbapenemase genes (Li et al., 2024).

The *bla*_KPC_ gene was initially linked to the 10 kb Tn*4401* transposon in the United States, which contains a transposase gene (*tnpA*), a resolvase gene (*tnpR*), and two insertion sequences (ISs), IS*Kpn6* and IS*Kpn7*, in addition to the *β*-lactamase gene *bla*_KPC-2_, which is highly mobile, enabling the gene to spread rapidly within bacterial populations (Abril et al., 2019; Hu et al., 2019; Naas et al., 2013)[8]. In China, however, the *bla*_KPC-2_ variant is frequently found within the Tn*1721* transposon, which shares similar transposase and resolvase genes but is distinct in its structure and mobility (Li et al., 2015; Shen et al., 2016; Yuan et al., 2021). Additionally, Tn*1403*, a transposon identified in *Pseudomonas* species, plays a significant role in the dissemination of resistance genes and is categorized within the Tn*21* subfamily of the Tn*3* family (Stokes et al., 2007; Vezina and Levesque, 1991). Separated by an identical backbone, the accessory regions of these four plasmids were composed of two IS*26*-associated modules, namely, the IS*26*-*bla*_KPC-2_-IS*26* and IS*26*-Tn*6376*-IS*26*, which originated from Tn*6296*. The *bla*_KPC-2_ gene in Tn*6296* was flanked by IS*Kpn27* and IS*Kpn6*, followed by the *korC*-*orf6*-*klcA*-*repB* gene (Fang et al., 2023).

The *bla*_KPC_ gene is predominantly disseminated in PA via transposition, frequently co-existing with other ISs or transposases, thereby creating a complex antibiotic resistance gene landscape. Clonal lineages such as ST463 are notably prevalent in specific geographical areas, with *bla*_KPC_-harboring strains typically exhibiting heightened resistance and virulence. In this study, we described the multidrug resistance and genomic features of *bla*_KPC-2_-positive PA isolate (PAE3), belonging to ST463, and integrated 75 plasmid-borne *bla*_KPC_ PA strains from the public database to demonstrate the mobilized platform characterization of the *bla*_KPC_ gene in PA. This comprehensive analysis aims to enhance our understanding of the genetic mechanisms underlying the development and global spread of carbapenem resistance in PA, with important implications for public health and antimicrobial resistance management.

## Materials and methods

### Bacterial collection

We used the specific primers KPC-F: TGTCACTGTATCGCCGTC and KPC-R: CTCAGTGCTCTACAGAAAACC to screen for 100 CRPA strains and identified the PAE3 strain that produces KPC. The strain PAE3 was obtained from the sputum sample of a 90-year-old patient with severe pneumonia in the intensive care unit of the Second Affiliated Hospital of Guangzhou Medical University in November 2023. It was identified using the VITEK-2 COMPACT automatic microbial identification system (bioMérieux, Marcy-l’Étoile, France).

### Antimicrobial susceptibility test

Antimicrobial susceptibility testing of PAE3 was performed using the broth microdilution method, which contained 50 μL of drugs, and 50 μL inoculum of 5 × 10^5^ CFU/mL concentration was added to 96 well plates and incubated in Mueller Hinton Broth at 35 °C for 18 h. The antimicrobial agents included ceftazidime, cefepime, imipenem, meropenem, ciprofloxacin, levofloxacin, amikacin, tobramycin, colistin, ceftazidime–avibactam, piperacillin-tazobactam, and ticarcillin-clavulanic acid. The results for colistin were confirmed according to EUCAST SOP 10.2, and the other results were interpreted based on the CLSI 2025 M100 (Table 1).

**Table 1:** Minimum inhibitory concentration (MIC) values of PAE3.

Antibiotics		MIC (μg/mL)	Interpretation	Interpretive categories of MIC (μg/mL)			Standard reference document
Categories	Name			Susceptible (S)	Intermediate (I)	Resistance (R)	
Cephalosporins	Ceftazidime	16	I	≤4	8	≥16	CLSI 2025 M100
	Cefepime	≥32	R	≤2	4–8	≥16	CLSI 2025 M100
Carbapenems	Imipenem	≥16	R	≤1	2	≥4	CLSI 2025 M100
	Meropenem	≥16	R	≤1	2	≥4	CLSI 2025 M100
Quinolones	Ciprofloxacin	≥4	R	≤0.25	0.5	≥1	CLSI 2025 M100
	Levofloxacin	≥8	R	≤0.5	1	≥2	CLSI 2025 M100
Aminoglycosides	Amikacin	≤2	S	≤4	8	≥16	CLSI 2025 M100
	Tobramycin	≥16	R	≤2	4	≥8	CLSI 2025 M100
Polymyxins	Colistin	2	S	≤2		≥4	EUCAST SOP 10.2
β-lactam/β-lactamase inhibitor combinations	Piperacillin-tazobactam	≥64/4	R	≤8/4		≥32/4	CLSI 2025 M100
	Ticarcillin-clavulanate	≥128	R	≤16/2	32/2–64/2	≥128/2	CLSI 2025 M100

S, susceptible; R, resistant; I, intermediate.

### Whole genome sequencing and bioinformatics analysis

Whole genome sequencing of PAE3 was performed using paired-end sequencing with Illumina Novaseq (2×150 bp paired-end reads), and long sequencing was performed with PacBio Sequel II by Shanghai Biozeron Biotechnology Co., Ltd. (Shanghai, China). PacBio reads were assembled using Unicycler v0.4.8 (https://github.com/rrwick/Unicycler) and polished by Pilon v1.22m (https://github.com/broadinstitute/pilon) with default parameters (Walker et al., 2014). The assembled genome of *P. aeruginosa* strain PAE3 was submitted to the NCBI GenBank database and annotated using the NCBI Prokaryotic Annotation Pipeline (Sayers et al., 2020; Tatusova et al., 2016). For PAE3 and other KPC-PA strains, multilocus sequence typing (MLST) was performed using MLST v2.0 (https://cge.food.dtu.dk/services/MLST/) (Larsen et al., 2012). Antimicrobial resistance genes (ARGs) and plasmid incompatibility types were identified using ResFinder v4.6.0 (http://genepi.food.dtu.dk/resfinder) (Bortolaia et al., 2020) and PlasmidFinder v2.1 (https://cge.food.dtu.dk/services/PlasmidFinder/), (Carattoli et al., 2014), respectively, with default thresholds. The oriTfinder2 (https://tool2-mml.sjtu.edu.cn/oriTfinder/oriTfinder) (Liu et al., 2025) was utilized to predict plasmid mobility by identifying the origin of transfer (*oriT*) and other related components under the default parameters..

### Genetic environment analysis of *bla*_KPC_ in PA

We performed MegaBLAST (https://doi.org/10.1093/bioinformatics/btn322) analysis of the *bla*_KPC-2_ gene sequence against the GenBank nonredundant (nr) *P. aeruginosa* (taxid: 287) database (on 23 April 2024) to identify 75 KPC-PA isolates harboring *bla*_KPC_, applying thresholds of 100.00% coverage and > 99.00% identity (Supplementary Table S1). The variants of *bla*_KPC_ genes were further analyzed using the CARD database (Alcock et al., 2023) and the BLDB database (Naas et al., 2017). The ISs and transposons (Tn) adjacent to the ARGs were identified using ISfinder (Siguier et al., 2006) with default parameters. Easyfig 2.2.5 (Sullivan et al., 2011) was used to visualize the genetic context of *bla*_KPC_ genes. The presence/absence of orthologous gene families of the *bla*_KPC_-positive plasmids in PA was analyzed using the phylogenetic cladogram. A binary gene presence/absence matrix was built using Orthofinder 2.5.4 (Emms and Kelly, 2019) with predefined parameters and visualized through iTOL 6.8.1 (Letunic and Bork, 2021).

## Results

### Antibiotic resistance profiles and genomic characterization of PAE3

Antimicrobial susceptibility testing results highlighted that PAE3 exhibited resistance to cephalosporins (Cefepime), carbapenems (imipenem and meropenem), quinolones (ciprofloxacin and levofloxacin), aminoglycosides (amikacin and tobramycin), and β-lactam/β-lactamase inhibitor combinations (piperacillin-tazobactam and ticarcillin-clavulanate). In addition, it exhibited intermediate resistance to ceftazidime and susceptibility to colistin and ceftazidime-avibactam (Supplementary Table S1).

**Figure 1:**
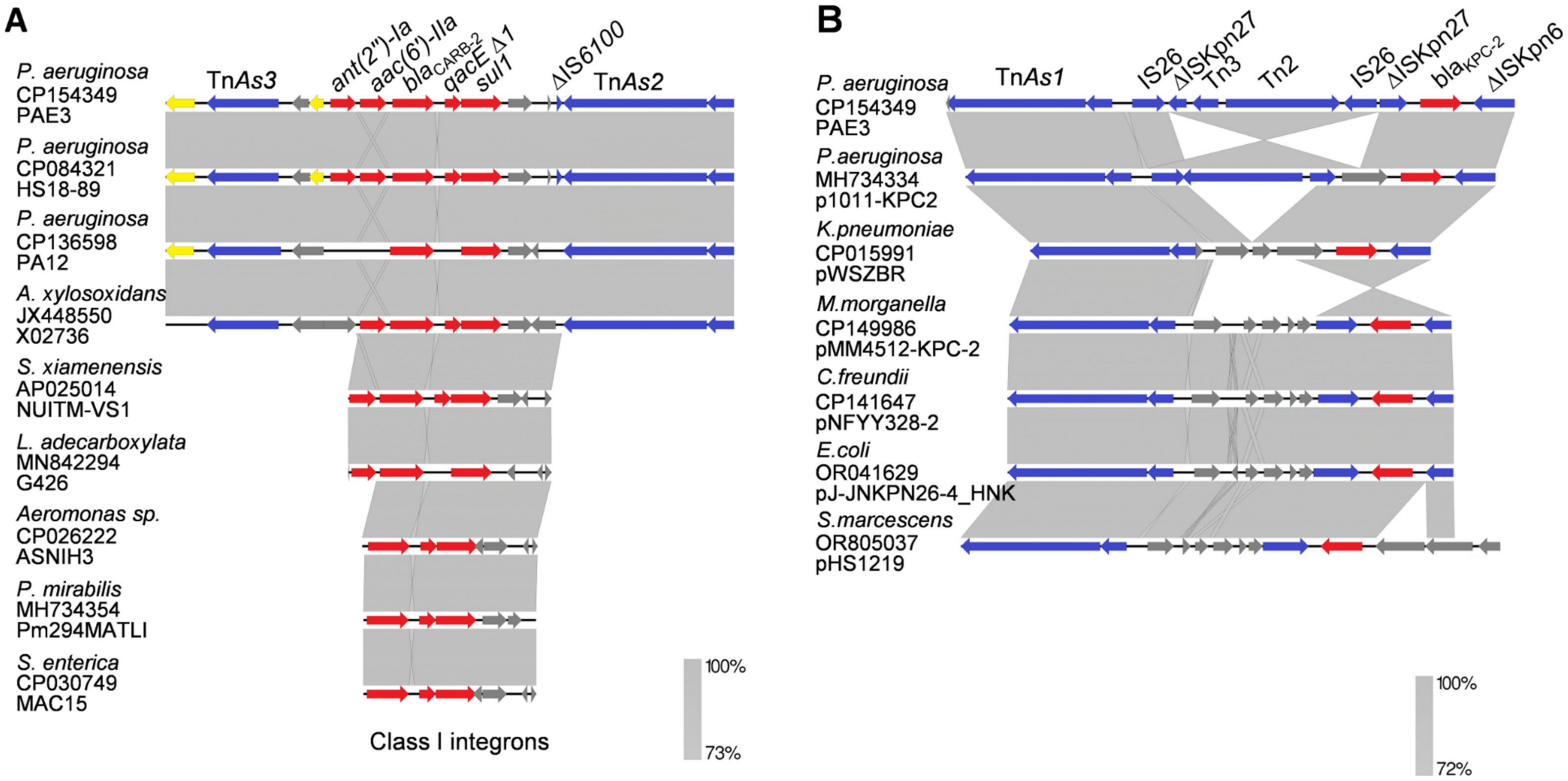
Sequence comparison of the genetic environment in PAE3 and other species. The shaded area between the sequences delimits the alignment regions with a percentage identity of ≥ 72%. **(A)** The regions surrounding *intI1* in Tn*6203*. **(B)** The regions surrounding the chromosome-borne *bla*_KPC-2_ gene in Tn*1721*. Gray shading represents a homology region. The red, blue, and gray arrows indicate resistance genes, mobile elements, and other open-reading frames, respectively.

**Figure 2:**
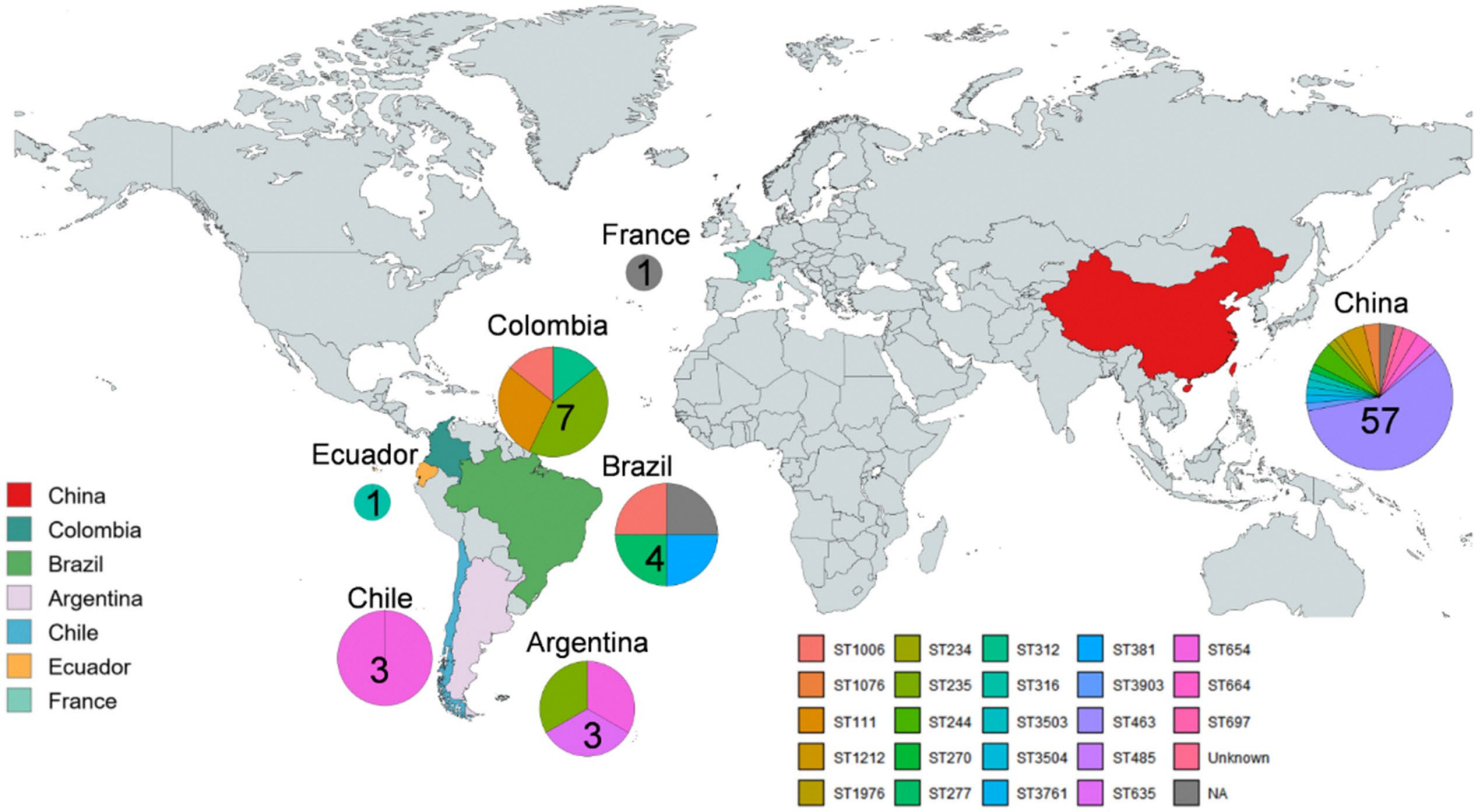
Geographic distribution of the *bla*_KPC_-harboring CRPA strains with known sequence types. The color shading represents the country with KPC-PA strains. Pie charts refer to the proportion of representative sequence types of KPC-PA strains per country.

The complete sequenced genome of PAE3 consists of a 7,073.5 kb chromosome and a 3,292-bp plasmid pPAE3. Key genomic features include the following: a total of 6,617 genes (with 6,459 coding genes) and 6,537 coding sequences (CDSs) in total (6,537 of which encode proteins); a GC content of 65.79%; an N50 contig length of 8,889 bp; and an average sequencing depth of 399.3×. MLST analysis revealed that PAE3 belonged to ST463. PAE3 harbored chromosome-borne ARGs, including *ant(2″)-Ia*, *aac(6′)-IIa*, *bla*_CARB-2_, *sul1*, *bla*_KPC-2_, *bla*_PAO_, *aph(3′)-IIb*, *bla*_OXA-486,_ and *crpP* (Figure 1). However, the plasmid pPAE3 did not carry acquired ARG genes. The small plasmid pPAE3 was characterized as mobilizable by oriTfinder2 analysis, which detected a 38-bp *oriT* (coordinates 3173.0.3210) and an associated relaxase gene belonging to the MOB_V_ family. There is a multidrug-resistant (MDR) region located on the chromosome, which contains *intI1* with resistance gene cassettes *ant(2″)-Ia*-*aac(6′)-IIa*-*bla*_CARB-2_-*qacE∆1*-*sul1* flanked by Tn*As3* and *∆*IS*6100*-Tn*As2* (Figure 2A). This MDR region displayed 100.00% coverage and 99.78% identity of the segment in HS18-89 PA (CP084321), which is also present in other bacterial species such as *Achromobacter xylosoxidans*, *Shewanella xiamenensis,* and *Leclercia adecarboxylata*. The *bla*_KPC-2_ gene was located on a Tn*1721*-mediated~10 kb transposition unit, with IS*26*-*∆*IS*Kpn27*-Tn*3*-Tn*2*-*∆*IS*Kpn27* located upstream and truncated *∆*IS*Kpn6* located downstream of *bla*_KPC-2_ (Figure 2B), which was nearly identical to plasmid p1011-KPC2 (100.00% coverage and 99.78% identity). According to current knowledge, PAE3 is the first reported Tn*1721* transposon harbored on the chromosome of PA ST463, identified in South China.

### Overview of the 76 KPC-PA strains

In total, 75 KPC-PA with clear genetic location (plasmid or chromosome) were used to conduct further analysis (Table 2). For some KPC-PA strains with incomplete data, we supplemented information on their ST types and geographic distribution by consulting relevant literature. All data used in this study were derived from authentic sequences in the NCBI database that provided reliable and accurate analysis information. The isolation dates of a total of 76 KPC-PA strains spanned from 2006 to 2024. Since 2015, the detection rate of KPC-PA has significantly increased and is closely associated with the transmission of mobile genetic platforms carried by this clonal strain.

**Table 2:** Summary of the *bla*_KPC_ genetic context in 76 KPC-PA.

Numbers of strains	Genetic structure	Variants of *bla*_KPC_	MLST	Country	Numbers in chromosome	Numbers in plasmid	Copies in one strain
15	Tn*1403*	*bla*_KPC-2_, *bla*_KPC-3_, *bla*_KPC-33_, *bla*_KPC-113_	ST463, ST485, ST244, ST664, ST1076, ST3504, ST3761, ST1212, ST3903	China	2	13	1
10	Tn*1721*	*bla*_KPC-2_	ST463	China	5	5	1
11	Tn*4401*	*bla*_KPC-2_, *bla*_KPC-3_, *bla*_KPC-31_, *bla*_KPC-33_	ST111, ST312, ST235, ST654	Argentina, Chile, Brazil, France, Colombia, Ecuador	4	7	1 or 2
8	Tn*6296*-like	*bla*_KPC-2_	ST463	China	0	8	1 or 2
14	IS*26*-*∆*IS*Kpn27*-*bla*_KPC-2_-*∆*IS*Kpn6*	*bla*_KPC-2_, *bla*_KPC-33_, *bla*_KPC-90_	ST463	China	6	8	1–5
6	*∆*IS*Kpn6*-*bla*_KPC-2_-Tn*3*-IS*Kpn27*-Tn*3*-Tn*2*	*bla*_KPC-2_, *bla*_KPC-87_	ST697, ST235, ST270, ST635	China, Argentina, Colombia	0	6	1 or 2
3	IS*26*-*∆*IS*26*-Tn*3*-IS*Kpn27*-*bla*_KPC-2_-IS*26*	*bla*_KPC-2_	ST463	China	0	3	1
13	Others	*bla*_KPC-2_	ST234, ST244, ST316, ST381, ST463, ST1006, ST1976, ST3503	Brazil, Colombia, China	0	13	1 or 2

**Figure 3:**
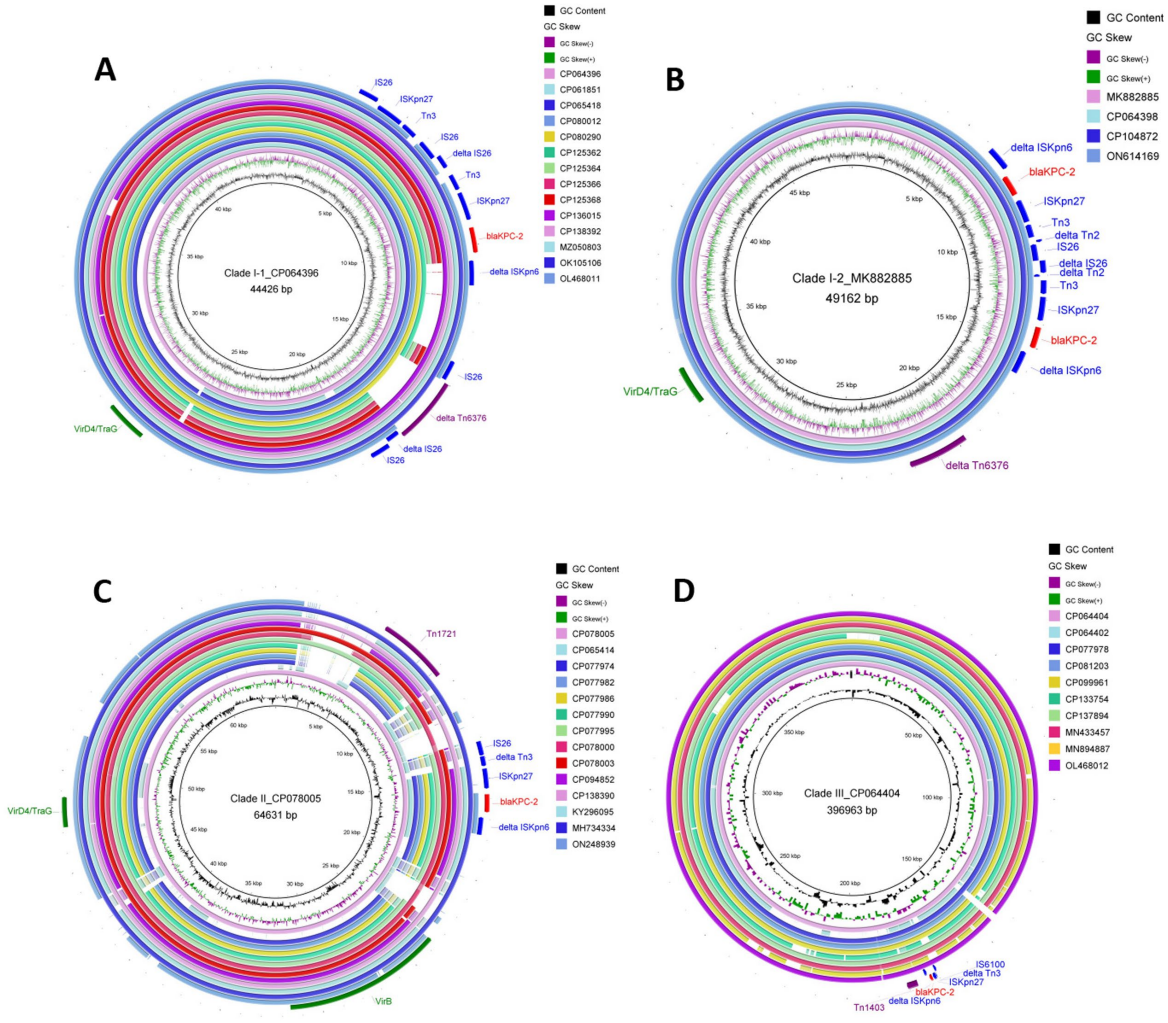
Comparative analysis of plasmids harbored in clades I to III. **(A)** Characteristics of the genetic environment of *bla*_KPC_ carried by the plasmids belonging to clade I. **(B)** Details of the 2-copies-*bla*_KPC_ genetic structures identified in 4 plasmids, which are clustered into clade I. **(C)** An overview of the genetic environment surrounding *bla*_KPC_ carried by plasmids in the clade II cluster. **(D)** Genetic structure of *bla*_KPC_-positive plasmids classified into the clade III cluster. Resistance, transposase, and genes encoding type IV coupling protein (T4CP) are shown in red, blue, and green arrows, respectively. The purple shades denote transposons. Arrows indicate the translation orientation of the coding genes.

Among total strains, *bla*_KPC-2,_ detected in 81.9% strains, was the most prevalent variant, followed by *bla*_KPC-3_ (*n* = 3), *bla*_KPC-33_ (*n* = 2), *bla*_KPC-87_ (*n* = 1), *bla*_KPC-90_ (*n* = 1), and *bla*_KPC-113_ (*n* = 1). The global dissemination of KPC-PA exhibits distinct geographic patterns Figure 2). The major countries included China (*n* = 57, 75%), followed by Colombia (7, 9.2%), Brazil (4, 7.0%), Argentina (3, 5.3%), and Chile (3, 5.3%) (Figure 3). A total of 24 distinct STs were identified, with ST463 (33/76), ST235 (4/76), and ST654 (4/76) accounting for the top three STs. The ST463 KPC-PA lineage was the most prevalent genotype in China (57.9%), and ST654 was prevalent in South American countries such as Argentina, Brazil, and Chile. The ST235 and ST311 lineages were primarily reported in Colombia and ST66 in France. Certain STs (e.g., ST4 and ST48) appear globally distributed, whereas others (e.g., ST1070 in China and ST277 in Brazil) are regionally restricted.

### Genetic mapping of the *bla*_KPC_ gene in PA

In 76 KPC-PA isolates, 13 strains (from No.1 to No.13 in Supplementary Table S1) contained the chromosome-borne *bla*_KPC_ gene. A total of 5 strains (38.5%) were associated with a Tn*1721* transposon variant, 4 strains (30.8%) with a Tn*4401* structure, and 2 strains (15.4%) with a Tn*1403* transposon. PAE3 and the other 4 KPC-PA strains containing Tn*1721*-like transposons (NDTH9845, NDTH7329, HS18-89, and PA12) all belong to the ST463 PA from China. In total, 2 strains harboring Tn*1403* transposons (SRRSH15 and SRRSH1521) belong to the ST244 from Hangzhou, China, and 4 strains, 24Pae112, M27432, 34Pae36, and HDR5_1, containing Tn*4401* transposons, belong to ST235 (Colombia), ST235 (Argentina), ST111 (Colombia), and ST316 (Ecuador), respectively.

**Figure 4:**
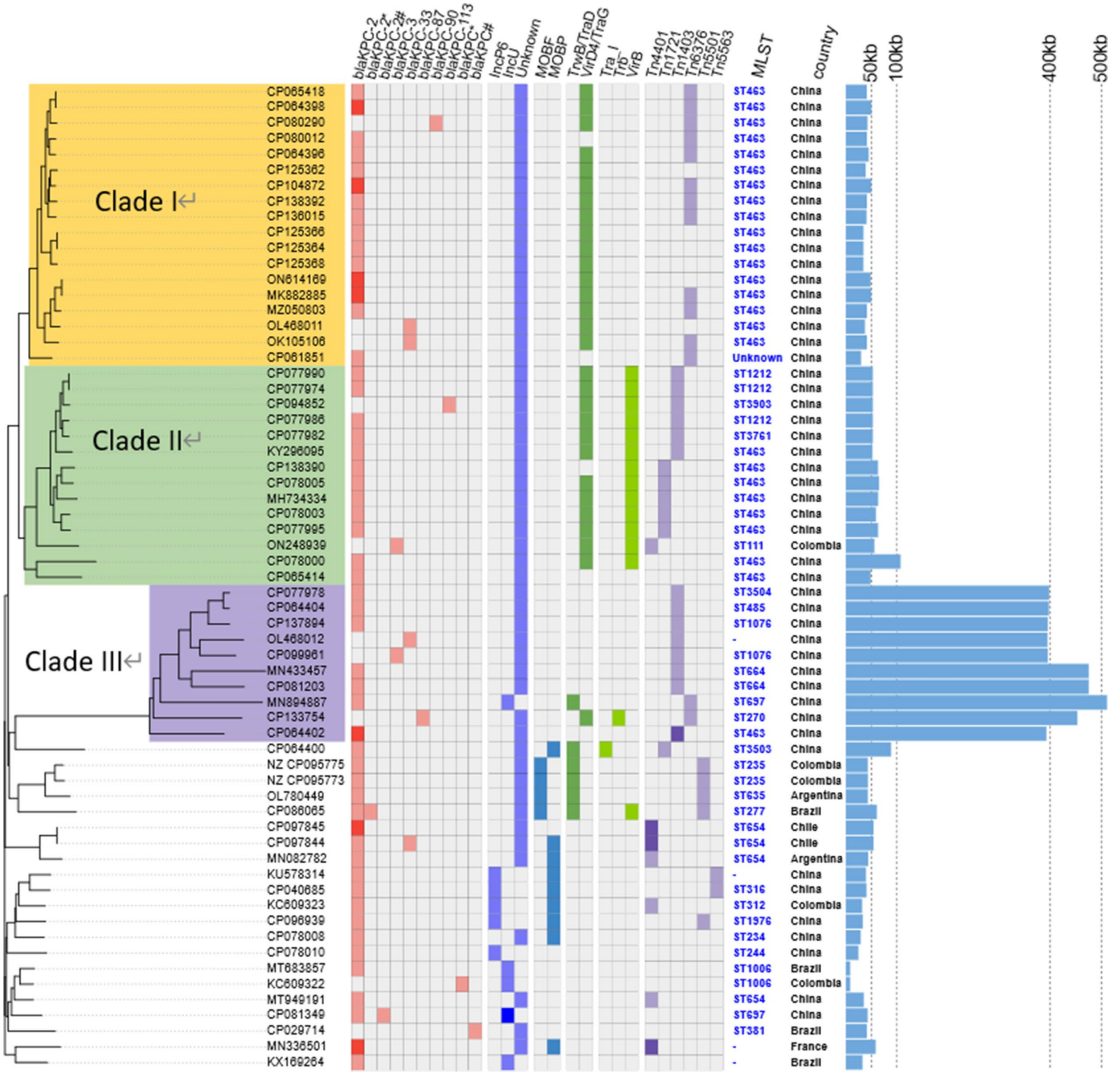
Details of variants of *bla*_KPC_ genes, replicon types, conjugative transfer regions, transposon classification, MLST, and geographical and length distribution of the 63 *bla*_KPC_-harboring plasmids in KPC-PA. The three major categories of information present in this figure include the phylogenetic tree, variants of *bla*_KPC_, replicon types, conjugative transfer regions (oriT, relaxase, T4CP, and T4SS), transposon classification, MLST, and geographical and length distribution of the 63 *bla*_KPC_-harboring plasmids of KPC-PA. A subset of these 63 plasmids can be divided into three major evolutionary groups and referred to as clade I, II, and III.

In total, 63 strains (from No.14 to No.76 in Supplementary Table S1) harbored plasmid-mediated *bla*_KPC_ gene; 5 strains (7.94%) contained elements corresponding to a Tn*1721* transposon variant; 7 strains (11.1%) aligned with the Tn*4401* transposon; 13 strains (20.6%) indicated *bla*_KPC_ within a Tn*1403* structure; 8 strains (12.7%) harbored a Tn*6296*-like transposon; and 1 plasmid (pCCBH28525_KPC) was associated with Tn*5501* transposon (Supplementary Table S1) (Silveira et al., 2022). The phylogenetic tree of the 63 *bla*_KPC_-harboring plasmids demonstrated that most isolates were clustered into three primary clades and named as I–III (Figure 4).

Clade I comprised 18 plasmids, ranging from 29.40 to 44.43 kb (Figure 4). These plasmids were mainly identified in ST463 and geographically distributed in China. Four copies of *bla*_KPC-2_, two *bla*_KPC-33,_ and one *bla*_KPC-90_ harboring plasmids were also classified into clade I. For the conjugative transfer regions, the genes encoding T4CPs of VirD4/TraG subfamily were characterized by the domain “TrwB_AAD_bind (PF10412)”but no relaxase and T4SS gene clusters were identified, inferred to be putative non-conjugative plasmids (Figure 3). The *bla*_KPC_ gene was mainly located on the composite transposon “IS*26*-IS*Kpn27*-Tn*3*-IS*26*-*∆*IS*26*-Tn*3*-IS*Kpn27*-*bla*_KPC_-*∆*IS*Kpn6*” and two copies of *bla*_KPC-2_ harboring four plasmids comprised the transposon “*∆*IS*Kpn6*-*bla*_KPC-2_-IS*Kpn27*-Tn*3*-*∆*Tn*2*-IS*26*-*∆*IS*26*-*∆*Tn*2*-Tn*3*-IS*Kpn27*-*bla*_KPC-2_-*∆*IS*Kpn6*” (Figure 1 and Table 2).

Clade II included 12 *bla*_KPC-2_-harboring, 1 *bla*_KPC-3_-harboring, and 1 *bla*_KPC-113_-harboring plasmids, with lengths ranging from 48.31–106.7 kb (Figure 4). Major plasmids carried single ARG and genes encoding T4CP or T4SS, while no genes related to the release of the MOBP family were detected (Figure 3). Plasmids in clade II were not mobilizable according to the structure of conjugative transfer regions. In total, 13 plasmids (92.86%) were geographically found in China, which included ST463, ST1212 (*n* = 3), ST11 (*n* = 1), ST3761 (*n* = 1), and ST3903 (*n* = 1). The *bla*_KPC_ gene located on plasmids of clade II was characterized by “IS*Kpn27*-*bla*_KPC_-*∆*IS*Kpn6*” (Figure 3).

Clade III comprised 10 MDR plasmids with lengths ranging from 392.2 to 510.711 kb, which were only discovered in China, including *bla*_KPC-2_-harboring, *bla*_KPC-3_-harboring, *bla*_KPC-33_-harboring, and *bla*_KPC-87_-harboring plasmids (Figure 4 and Supplementary Table S2). All plasmids contained only one single-replicon, one plasmid with IncU replicons, and the other plasmids with unknown replicons. Most plasmids belonging to clade III had no genes encoding relaxase, T4CP, or T4SS, inferred to be putative non-conjugative plasmids (Figure 3).

### Genetic platforms mobilizing *bla*_KPC_ gene in PA

#### *bla*_KPC_ within Tn*1403* transposon in KPC-PA

**Figure 5:**
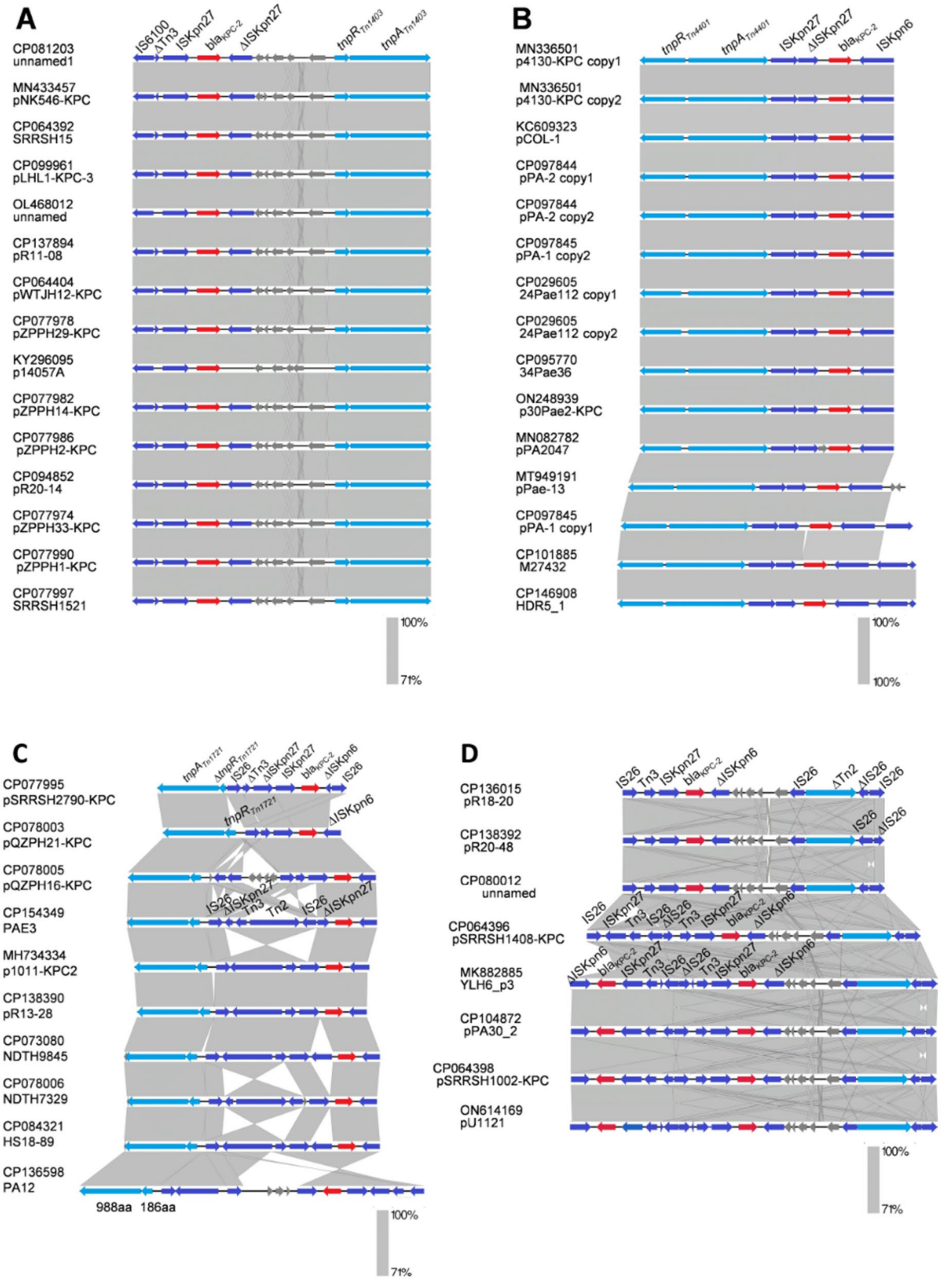
Sequence comparison of four distinct transposons surrounding the *bla*_KPC_ gene in CRPA. **(A)** The genetic environment of the Tn*1403* transposon. **(B)** The genetic environment of the Tn*4401* transposon. **(C)** The genetic environment of the Tn*1721* transposon. **(D)** The genetic environment of the Tn*6296*-like transposon. The red, light blue, blue, and gray arrows indicate *bla*_KPC_ gene, transposases, transposable elements, and other open-reading frames, respectively.

A total of 15 *bla*_KPC_ MGEs from 15 strains were classified into the Tn*1403* transposon, including 2 from the chromosome and 13 contained in plasmids. Members of this group were narrowly geographically distributed in China and belonged to ST244, ST463, ST485, ST664, ST1076, ST3504, ST3761, ST1212, and ST3903 (Table 2). Among all KPC-PA strains, there were 12 *bla*_KPC-2-_, 1 *bla*_KPC-3_, 1 *bla*_KPC-33_, and 1 *bla*_KPC-113_ harboring strains, which were classified as part of the Tn*1403* cluster. Notably, each strain harboring Tn*1403* transposons carried exactly one copy of the *bla*_KPC_ gene. We explored the genetic environment surrounding the *bla*_KPC_ genes located on the composite transposon Tn*1403*, which comprises a highly conserved region “IS*6100*-*∆*Tn*3*-IS*Kpn27*-*bla*_KPC-2_-*∆*IS*Kpn27*,” as well as four additional elements upstream of the *tnpR* and *tnpA* (Figure 5A).

#### *bla*_KPC_ within Tn*1721*-like transposons harboring in KPC-PA

The *bla*_KPC-2_ MGEs from 10 strains were classified into the Tn*1721* cluster, which exhibited an equal division between chromosomes and plasmids (Table 2). The PAE3 strain also contained the *bla*_KPC-2_ gene with the composite transposon Tn*1721* (Figure 5C). We explored the genetic context associated with the *bla*_KPC-2_ gene within Tn*1721*, which carried the conserved structure of “tnpA_Tn*1721*_-tnpR_Tn*1721*_-IS*26*-*∆*Tn*3*-IS*Kpn27*-*bla*_KPC_-*∆*IS*Kpn6.*” Notably, all the strains classified into the Tn*1721* group were detected in China and belonged to ST463. In the KPC-PA, the combination of chromosomal carriage and Tn*1721* has been identified. The genetic environment of the *bla*_KPC_ gene is not totally identical, and the primary difference from the closest sequences is the inversion of Tn*3* and Tn*2*.

#### *bla*_KPC_ within Tn*4401* transposon in KPC-PA

Out of all the strains, the genetic structures surrounding *bla*_KPC_ (plasmid or chromosome) in 11 strains contain the Tn*4401*-like transposon. Among these isolates, 4 strains contained the *bla*_KPC_ gene in the chromosome and 7 strains harbored *bla*_KPC_ within a plasmid structure (Table 2). The genetic context surrounding the *bla*_KPC-2_ genes within Tn*4401* carried the conserved structure of “tnpA_Tn*4401*_-tnpR_Tn*4401*_-IS*Kpn7*-*∆*IS*Kpn7*-*bla*_KPC-2_-IS*Kpn6*” (Figure 5B). This genetic structure, commonly observed in ST235 and ST654, was widely distributed in Chile, Colombia, Argentina, and Ecuador (Table 2). Thus, the *bla*_KPC_ gene within Tn*4401* in PA was predominantly detected in Europe and the Americas, with a rare report in Asia.

#### *bla*_KPC-2_ within Tn*6296*-like transposon in KPC-PA

The *bla*_KPC-2_ genetic platform was identified in 8 strains of Tn*6296*-like transposon in China (Figure 4D). All *bla*_KPC-2_ genes were plasmid-borne and carried by ST463 PA. Additionally, 4 strains contained 2 symmetrically arranged copies of *bla*_KPC-2_ (Table 2 and Figure 5D).

#### The genetic context of *bla*_KPC_ lacking a typical transposon structure in a plasmid

**Figure 6:**
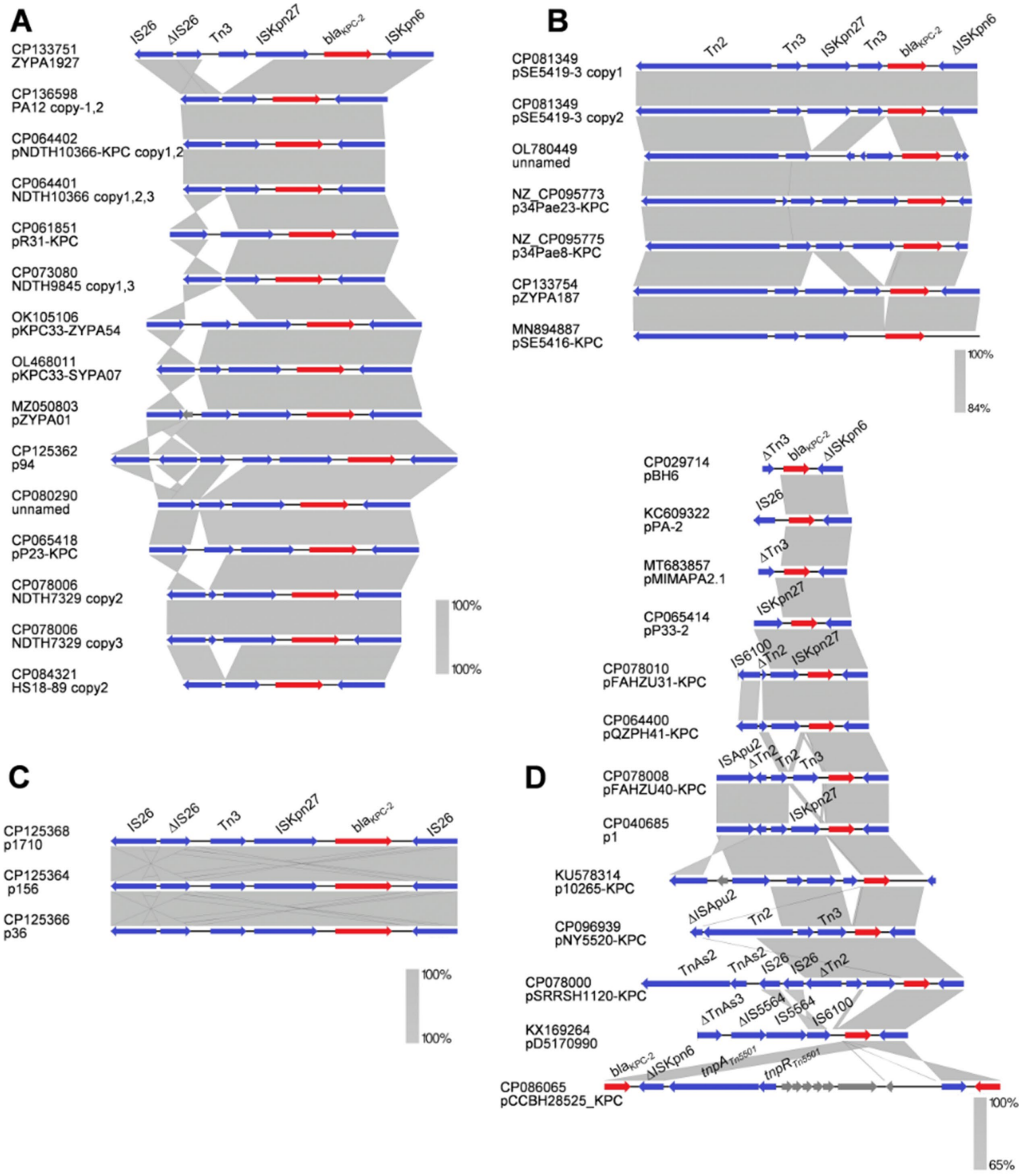
Sequence comparison of transposons surrounding the *bla*_KPC_ gene in CRPA, which were not compared to the typical transposons compiled in this study. **(A)** The genetic environment of *bla*_KPC_ in “IS*26*-Tn*3*-IS*Kpn27*-*bla*_KPC-2_-IS*Kpn6*” structure. **(B)** *bla*_KPC_ in “Tn*2*-Tn*3*-IS*Kpn27*-Tn*3-bla*_KPC-2_-*∆*IS*Kpn6*” structure. **(C)** *bla*_KPC_ in “IS*26*-*∆*IS*26*-Tn*3*-IS*Kpn27*-*bla*_KPC-2_-IS*26*.” **(D)** *bla*_KPC_ flanked with *∆*IS*Kpn6* and no typical structural classification. The shaded area of the four genetic context subgroups of the sequences delimits the alignment regions with 100% identity. **(A)** ≥ 84% **(B)** and 100% **(C)** and ≥65% **(D)**, respectively. The red, blue, and gray arrows indicate *bla*_KPC_ gene, mobile elements, and other open-reading frames, respectively.

In total, 14 strains harbored 21 *bla*_KPC-2_, 1 *bla*_KPC-33,_ and 1 *bla*_KPC-90_ genes, which were flanked by IS*Kpn6*/*∆*IS*Kpn6* and IS*Kpn27*-IS*26*, and no typical transposase enzymes were identified near *bla*_KPC_ (Figure 6A). This genetic structure exhibited high copy numbers within individual strains (with up to five identical copies) and was prevalent in ST463 PA in China (Table 2). A total of 6 strains carried distinct genetic *bla*_KPC_ gene clusters, characterized by a conserved genomic architecture with truncated IS*Kpn6* elements flanking an intact Tn*3*-Tn*2* transposon (Figure 6B). These strains are distributed in China, Argentina, and Colombia, which represent diverse STs, including ST270, ST697, ST235, and ST635 (Table 2). Moreover, 3 ST463 PA strains contained the *bla*_KPC-2_ gene within the structure “IS*26*-*∆*IS*26*-Tn*3*-IS*Kpn27*-*bla*_KPC-2_-IS*26*” and formed a distinct cluster in this study (Figure 6C and Table 2). There were still 13 strains that could not be classified into specific groups but shared the common feature of truncated IS*Kpn6* elements flanking the *bla*_KPC_ gene. Remarkably, the CCBH28525 strain from Brazil presented 2 copies of the *bla*_KPC_ gene flanking a central region containing transposase and resolvase genes of the Tn*5501* family (Figure 6D).

## Discussion

The emergence and spread of MDR bacteria in recent years have posed significant challenges to the clinical management of bacterial infections. *IntI1* commonly harbors multidrug resistance gene cassettes and can be transferred via mobile genetic elements, with the incidence ranging from 22 to 59% (Liu et al., 2020). The *intI1* is frequently linked to resistance genes, including *ant(2″)-Ia*, *aac(6′)-IIa*, *bla*_CARB-2_, *qacE∆1*, and *sul1*, which are flanked by transposons (Tn*As3* and *∆*IS*6100*-Tn*As2*) and integrated into the chromosome of *P. aeruginosa* (Liu et al., 2020). The conservation of specific gene clusters across bacterial species suggests the occurrence of horizontal gene transfer, which facilitates the spread of antibiotic resistance (Deng et al., 2015; Gillings et al., 2008).

The global spread of carbapenem resistance among Gram-negative bacteria is driven by the horizontal transfer of resistance genes via active transposons and diverse plasmids, including *bla*_OXA-232_, *bla*_IMP-4_, and *bla*_VIM-71_ (Heng et al., 2024; Zheng et al., 2024; Zheng et al., 2023). This mechanism facilitates the rapid dissemination of carbapenemases such as *bla*_KPC_, contributing to their widespread prevalence in different regions (Galetti et al., 2016; Hu et al., 2024a). However, the *bla*_KPC_ gene exhibited similar transmission patterns between carbapenem-resistant KP and PA, while the primary differences were observed in clonal diversity, regional prevalence, resistance gene profiles, and clinical symptoms. The dissemination of the *bla*_KPC_ gene in KP typically exhibits a broader geographical distribution, whereas KPC-PA specifically demonstrates a regional prevalence. In the Pearl River Delta region, the presence of plasmid-mediated KPC-PA has only been documented by Professor Chen Dingqiang from Zhujiang Hospital in 2021 (Wang et al., 2021). To the best of our knowledge, this study is the first to identify chromosomally mediated KPC-PA in South China, representing a significant advancement in the understanding of this pathogen (Wu et al., 2021; Yuan et al., 2021; Zhang et al., 2022). We conducted a comprehensive genomic investigation of the carbapenem-resistant PAE3 strain, which carries the *bla*_KPC-2_ gene, and characterized the genetic determinants mediating horizontal transfer of resistance genes. To further understand the global dissemination of KPC-PA, we systematically analyzed *bla*_KPC_ gene variants, genetic contexts, and the distribution patterns of 76 KPC-PA strains, which vary across countries and span a broad temporal range (2006–2024). The results provided novel insights into: (1) the role of mobile genetic platforms in facilitating the *bla*_KPC_ gene in PA and (2) the molecular epidemiology of KPC-PA. Through our study, 7 distinct *bla*_KPC_ gene variants (*bla*_KPC-2_, *bla*_KPC-3_, *bla*_KPC-31_, *bla*_KPC-33_, *bla*_KPC-87_, *bla*_KPC-90_, and *bla*_KPC-113_), 4 predominant transposon types (Tn*1403*, Tn*1721*, Tn*4401*, and Tn*6296*-like), and 24 STs across seven different countries were discovered. The *bla*_KPC-2_ gene is commonly associated with the Tn*1721-*, Tn*1403-*, and Tn*6296*-like transposon structures in PA strains from Eastern China. In contrast, the spread of the *bla*_KPC-2_ gene in the Americas, Europe, and other regions was mediated by the Tn*4401* transposon (Hu et al., 2024b). Notably, the Tn*1721* transposon structure is uniquely detected in ST463 PA strains, which were integrated into both chromosomes and plasmids (Zhang et al., 2023). Our findings indicate that the *bla*_KPC_ gene carried by the Tn*1721* transposon in ST463 PA exhibits a high prevalence in China. Our study suggests that the *bla*_KPC_ gene harbored within the Tn*1721* transposon could contribute to the adaptability of PA against diverse environmental conditions across Eastern China, which is also predominantly found in *K. pneumoniae* and other *Enterobacteriaceae* in China (Hu et al., 2024b; Reyes et al., 2023; Tang et al., 2017). Furthermore, *bla*_KPC_-producing PA strains could be involved in the emergence and spread of multidrug resistance, potentially in combination with other carbapenem genes, including *bla*_VIM_ and *bla*_IMP_ (Fang et al., 2023; Tenover et al., 2022).

The Tn*3*-family transposon Tn*4401* is an important mobile genetic element of the *bla*_KPC_ gene and was identified in all isolates. Tn*1403* transposon was initially recovered from a MDR clinical PA strain recognized in the United States in 1973–1974 (Vezina and Levesque, 1991). The *tnpA* and *tnpR* genes of Tn*1403* exhibited 97.4 and 39.9% similarity to those of Tn*1721*, respectively. Tn*6296* is also a common transposon and is formed by the insertion of the “core *bla*_KPC_ platform” of Tn*1722*. This insertion truncates the *mcp* gene of Tn*1722* to an incomplete structure. The genetic environment of Tn*6296* is modulated by elements such as IS*26*, which may lead to more complex changes in the transposon structure related to the *bla*_KPC_ gene. In addition, Tn*6296* exhibits structural conservation with other transposons such as Tn*3*, but harbors distinct features for the dissemination and evolution of resistance genes (Yuan et al., 2021). IS*26* contributes to the mobilization of resistance genes into the gene pool by forming transposons (Harmer and Hall, 2016; Tang et al., 2024). The presence of IS*26* in this study is notable, as it could mediate diverse mobilization mechanisms. Specifically, IS*26* has been shown to facilitate the mobilization of adjacent genes independent of transposase enzymes, potentially reshaping intergenic regions (Harmer and Hall, 2016; Li et al., 2023). In MDR PA strains, IS*26* is predominantly present as multiple copies, with directly oriented elements forming pseudocomposite transposons (PCTns). IS*26,* belonging to the IS*6* family, often appears as multiple copies in the MDR PA strains, which, when oriented, form a composite transposon called pseudocomposite transposon (PCTn) and facilitate horizontal gene transfer through cointegration of donor and recipient DNA (Tang et al., 2024).

In response to the crisis of KPC-PA, a new generation of *β*-lactam/β-lactamase inhibitor combinations, including ceftazidime-avibactam, imipenem-relebactam, and meropenem-vaborbactam, have become vital therapeutic options. These agents target and inhibit KPC and effectively restore susceptibility by large retrospective analysis and ceftazidime-avibactam could inhibit PAE3 *in vivo* (Kang et al., 2024; Palomba et al., 2025; Sophonsri et al., 2024). There are several limitations here: our study focused only on *bla*_KPC_-positive PA but did not include other *Enterobacteriaceae*. Moreover, only the complete sequences of *bla*_KPC_-positive plasmids in KPC-PA from the NCBI database were selected for subsequent analysis, which may reflect a partial view of the real epidemiology. In summary, this research enhanced our understanding of the genetic mechanisms driving the global spread of *bla*_KPC_ in PA.

## Conclusion

The emergence and spread of PA pose significant clinical and epidemiological challenges. We identified a *bla*_KPC_-positive CRPA belonging to ST463, which is integrated into the chromosomal structure within a Tn*1721* transposon in this study. Additionally, we investigated the transposon structures contributing to the transmission of the *bla*_KPC_ gene in PA from a global perspective to improve the understanding of the mechanisms of KPC-PA. Our results suggest that the ST463 PA has evolved a clonal lineage and developed distinct genetic platforms that facilitate chromosomal integration of *bla*_KPC-2_ via Tn*1721*, a feature rarely reported in Southern China. Furthermore, our global analysis revealed three dominant genetic architectures driving *bla*_KPC_ dissemination: (1) Tn*1721* in Chinese ST463 PA; (2) Tn*4401* in ST235/ST654 PA from the Americas and Europe; (3) and Tn6296-like elements in plasmid-borne ST463 isolates. These findings underscore the role of mobile elements, including transposons and ISs, in the molecular epidemiology of the *bla*_KPC_ gene in PA.

## Importance

The emergence of carbapenem-resistant *Pseudomonas aeruginosa* (PA) that harbors the *bla*_KPC_ gene represents a critical threat to global public health, particularly due to limited treatment options and high mortality in vulnerable populations. This study was carried out to understand how mobile genetic elements drive the dissemination of *bla*_KPC_ in PA. To our knowledge, a clinically relevant ST463 PA strain (PAE3) carrying a chromosome-borne *bla*_KPC_ gene was first identified from southern China, which exhibited multidrug resistance. Combining a global dataset of 76 *bla*_KPC_-positive PA strains, the *bla*_KPC_ gene is predominantly disseminated through various transposon structures, including Tn*1721*, Tn*1403*, Tn*4401*, and Tn*6296*-like elements, which exhibit distinct regional distribution patterns. Tn*1721* is the dominant transposon found in Chinese ST463 PA, contrasting with Tn*4401*-mediated dissemination in ST235/ST654 PA observed in the Americas and Europe. This work provides novel insights into the mobilized genetic platforms that facilitate the dissemination of the *bla*_KPC_ gene in PA.

## Data Availability

The datasets presented in this study can be found in online repositories. The names of the repository/repositories and accession number(s) can be found at: https://www.ncbi.nlm.nih.gov/genbank/, CP154349.
